# Supplementary material for: Sex Hormone-Binding Globulin (SHBG) in Cerebrospinal Fluid Does Not Discriminate between the Main FTLD Pathological Subtypes but Correlates with Cognitive Decline in FTLD Tauopathies
Source: Biomolecules. 2021 Oct 8;11(10):1484. doi: 10.3390/biom11101484 (PMC8533538; doi:10.3390/biom11101484)
Supplement: Supplementary file 1 [file biomolecules-11-01484-s001.zip › biomolecules-1394965-supplementary.pdf]

Supplementary Figure S1

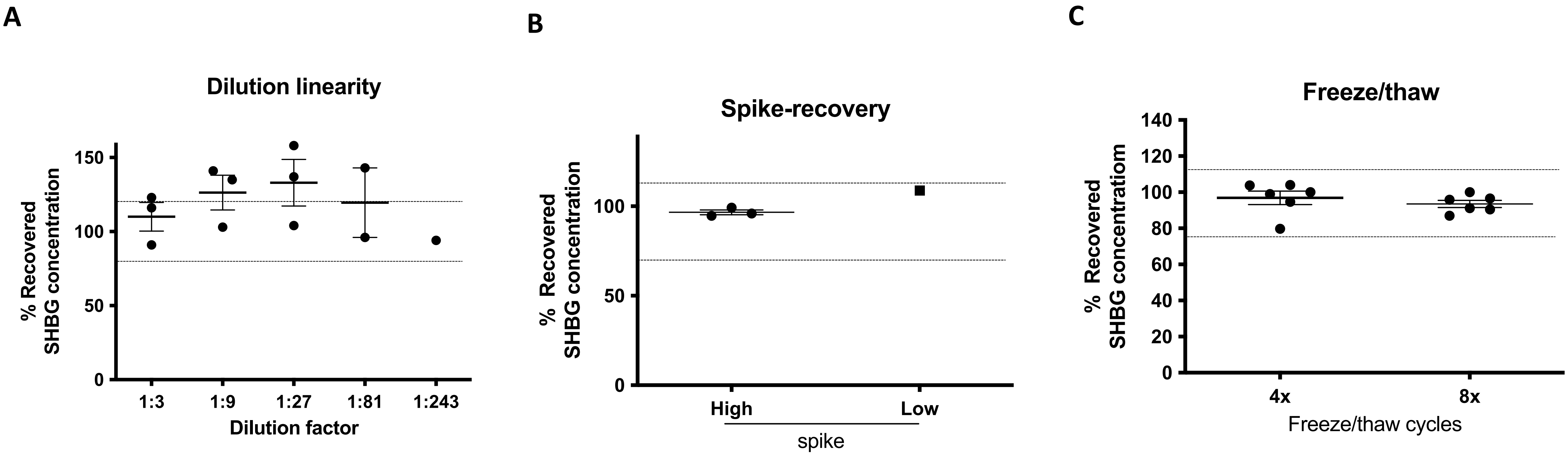

Supplementary Figure S1. SHBG ECLIA can optimally measure SHBG concentration in CSF. (A) For dilution linearity, three CSF samples were spiked with serum from a pregnant woman which contain high levels of SHBG. Samples were then serially diluted (3-fold) with assay dilution buffer. (B) For spike recovery, CSF samples were spiked with serum samples from a pregnant woman with known concentration of SHBG at two different dilutions (high and low). (C) For Freeze/thaw cycles, CSF samples underwent 2, 4 or 8 freeze thaw cycles. Thawing was for 2 hours at room temperature and freezing was at -80C minimally overnight. The sample with 2 freeze/thaw cycle was used as a reference sample. For all analysis, the % of recovered SHBG was calculated. Dotted lines represent the acceptable range (+ 20%). The mean and standard error of the mean are represented when appropriate.
